# CHILDHOOD DEPRESSION. Exploring the association between family violence and other psychosocial factors in low-income Brazilian schoolchildren

**DOI:** 10.1186/1753-2000-6-26

**Published:** 2012-07-09

**Authors:** Joviana Avanci, Simone Assis, Raquel Oliveira, Thiago Pires

**Affiliations:** 1 Jorge Careli Latin-American Center of Studies of Violence and Health (National School of Public Health) and Fernandes Figueira Institute/Oswaldo Cruz Foundation, Avenida Brasil 4036 sala 700, Manguinhos, Rio de Janeiro, CEP: 21040-361, Brazil; 2 Evandro Chagas Institute of Clinical Research/Oswaldo Cruz Foundation, Avenida Brasil 4036, Manguinhos, Rio de Janeiro, CEP: 21040-361, Brazil

**Keywords:** Depression, Children, Violence, Abuse

## Abstract

**Background:**

Childhood depression affects the morbidity, mortality and life functions of children. Individual, family and environmental factors have been documented as psychosocial risk factors for childhood depression, especially family violence, which results in inadequate support, low family cohesion and poor communication. This study investigates the association between psychosocial depression factors in low-income schoolchildren and reveals the potential trouble spots, highlighting several forms of violence that take place within the family context.

**Methods:**

The study was based on a cross-sectional analysis of 464 schoolchildren aged between 6 and 10, selected by random sampling from a city in the state of Rio de Janeiro, Brazil. Socio-economic, family and individual variables were investigated on the strength of the caregivers’ information and organized in blocks for analysis. A binary logistic regression model was applied, according to hierarchical blocks.

**Results:**

The final hierarchical regression analysis showed that the following variables are potential psychosocial factors associated with depression in childhood**:** average/poor relationship with the father (OR 3.24, 95% CI 1.32-7.94), high frequency of victimization by psychological violence (humiliation) (OR 6.13, 95% CI 2.06-18.31), parental divorce (OR 2.89, 95% CI 1.14-7.32) and externalizing behavior problems (OR 3.53 IC 1.51-8.23).

**Conclusions:**

The results point to multiple determinants of depressive behavior in children, as well as the potential contribution of psychological family violence. The study also reveals potential key targets for early intervention, especially for children from highly vulnerable families.

## Background

Depression affects the morbidity, mortality and life functions of children. Investigators are extensively discussing the rise of depression during the last decades in more recent cohorts [1]. Formal psychiatric diagnoses estimate that 0.3% to 7.8% of children under 13 years of age suffer from depression disorders [1-3]. Equally, in Brazil, the prevalence of depression in childhood is between 0.2% and 7.5% for children under 14, which varies mainly according to the assessment [4,5].

Depression in childhood is not simply a mood regulation disorder; it also involves alterations in the physiology and in the cognitive and social functions of children, and requires comprehension of developmental integration processes at multiple levels of biological, psychological and social complexity in individuals [6].

Individual, family and environmental factors have been documented as psychosocial factors for childhood depression. Individual factors include age, gender, psychological and physical vulnerability [7], comorbidity with other disorders [8], emotional disturbance, impaired sociability, low self-esteem and social skill difficulties [9,10]. Family factors associated with childhood depression vulnerabilities consist of child abuse and marital conflict [11,12]; parental depression [13]; rejection and low interaction with the child [14]; losses related to separation and death [15] and a history of insecure attachment [16,17]. Lastly, environmental factors include daily difficulties, stressful or traumatic life events [18,19], lack of social support and poor friendships [12-20].

Although there is evidence for the interaction of genetic and environmental factors in the development of depression in the prepubertal period, the genetic influence appears to be low and the disorder tends to be more strongly linked to environmental factors [21].

### Family Violence and Depression

Family violence is a worldwide public health problem of epidemic proportions. In worldwide terms, the statistics regarding the violent behavior of parents towards their children are around 23% [22-24]. In Brazil, the prevalence is between 10-15% [25,26]. There are high-risk factors for family violence, such as poverty, dysfunctional family life, substance abuse, and the vulnerability of some groups (males, ethnic minorities, and inner-city populations) [27]. Children exposed to these risk factors can be more vulnerable to the impact of traumatic events, due to their cumulative effects; also, they usually have less access to healthcare services, especially mental care. Burns et al. [28] explain that while approximately half of these vulnerable children are diagnosed with mental health problems, 75% of them do not receive treatment.

The short- and long-term effects of family violence on child development have been extensively studied. To better understand the effects, it is important to study the context in which family violence occurs, mainly the coexistence of intimate partner violence and child abuse [29-31]. Prior studies suggest that children are most affected by violence that impinges directly upon them. They may blame themselves and manifest feelings of shame, guilt, mistrust and low self-esteem [32]. Being a direct victim of violence can be worse than being a witness. Likewise, children may find it more stressful to observe violence between their parents than between strangers in the community. Nonetheless, the impact of witnessing violence in the home has a negative effect, since children may perceive the world as unsafe, adults as untrustworthy, and events as unpredictable or uncontrollable [33].

A violent family environment tends to engender low support, low cohesion and poor communication. Sharfstein [27] stated that the violence that affects children is the largest single preventable cause of mental illness: ‘*what cigarette smoking is to the rest of medicine, early childhood violence is to psychiatry*’ (p.2). The experience of violence triggers traumatic deregulations of neurobiological, cognitive, social, and affective processes that have different manifestations, depending on the child’s developmental stage [34].

Studies examining the relationship between family violence and depression have failed to take into account the impact of multiple psychosocial factors in the child´s life [35]. For example, in the analysis of the effects on children exposed to violence, it is important to check different types and levels of violence within intimate partner violence. It is also necessary to adopt a systemic approach in which the individual, family and social aspects are included [36], since, in less developed countries, child health is determined by a large number of factors. Thus, this work seeks to investigate the association of psychosocial factors of depression in low-income schoolchildren and reveal the potential factors, focusing on the various forms of family violence. The psychosocial factors are organized in levels, according to their impact, which range from the most proximal to the distal factors, which include the social and cultural context [37,38].

## Method

### Participants

The data are based on longitudinal research, which started in 2005 including 500 schoolchildren in the city of São Gonçalo in the state of Rio de Janeiro, Brazil [39]. São Gonçalo is a low-income city, located in the state of Rio de Janeiro, in the southeast of Brazil. It is the second-largest city in the state with a population density of 4.020. In 2011, São Gonçalo had a population of approximately 1 million people, 1/3 of them being children and adolescents. The city ranks in 995^th^ place in the Childhood Development Index in the country. Basic services including electricity, sanitation, and drinking water are not provided to the whole population, and residents have difficulty accessing healthcare services. Violence and accidents rank in fourth place among the causes of death in the city: 10.5% of all deaths in 2009.

This article consists of a cross-sectional analysis of the first wave of the longitudinal study (2005). The sample was collected among first grade students of the elementary public schools of the city. The multi-stage cluster sampling strategy involved a three-stage design, which included all 54 public schools, 236 first grade classes and 6.589 children. In the first stage, 25 schools were systematically selected with probability proportional to the size of the whole sample. In the other two-stages, two classes per school and ten students per class were selected by simple random sampling. Each child’s caregiver was invited for an interview. Frequent errors in the school lists (with names of children who never attended school) and the absence of the caregiver on the day scheduled for the interview (after three attempts) led to the replacement of about 35% of children initially sampled. In these cases, children were replaced by others until the total of ten students per class was attained. This sample design resulted in a systematic self-weighted sample, providing very little variation among the final survey weights.

The sample representation was examined by comparing the maternal educational level and family income of the sample with data on the whole adult population of the city studied. The mothers interviewed had lower educational levels than the women living in the city in general: 63.4% of the sample had less than 8 years of education as opposed to 56.5% among female residents. A similar difference was found regarding the average family income per month: approximately US$426.00 in the whole city and US$304.00 in the sample studied. Although the study found small differences, no bias was introduced. These differences were expected, since the sample investigated came exclusively from public schools, where the majority of low-income children study.

Eighty-four per cent of all caregivers interviewed were mothers, 4% fathers, 9% female relatives and 3% other people close to the family. Thirty-six children had an IQ ≤ 69 (Wechsler Intelligence Scale for Children III) [40] and children over 10 years old were excluded from this analysis.

The sample consisted of 464 schoolchildren ranging from 6 to 10 years old, with a mean age of 8 years. Out of the total number of children sampled, 52% were male; 66% were identified by the respondent as being black, 33% as being white, and 1% as being from another ethnic background. Only 13% of mothers and 17% of fathers had 11 or more years of education, and 42% of mothers and 14% of fathers were unemployed. With regard to income, 69% reported an income less than half a minimum wage (US$155) per capita. In terms of family structure, 54% of the children lived with both parents, 25% with one of the parents and stepparents, 17% with only one parent and 4% lived with other relatives.

The research project was authorized by the Human Research Ethics Committee of the Oswaldo Cruz Foundation, and written informed consent was obtained from all the children’s parents/legal guardians.

### Procedure and measures

The caregivers of the schoolchildren participated in a face-to-face interview, designed for gathering information about all the measures investigated: socio-demographic and family characteristics, and behavioral problems of the children including withdrawal/depression. All measures used referred to the lifetime of the child, with the exception of withdrawal/depression problems, which were applied to assess the last six months. The measures were divided into three main blocks, according to a hypothetic strength relation (distal to proximal impact) [40]. The distal block consists of the variables that rarely cause ill-health directly, the intermediate variables act through a number of inter-related factors and the proximate variables are those that may affect the outcome studied more directly [38]:

*Socio-demographic characteristics (distal level)* included the age and sex of the child and the social stratum, which was estimated according to the family´s assets and head-of-family´s schooling, scored as *upper/middle* and *lower social strata*[41].

*Family environment variables (intermediate level)* included the following variables: (1) family structure characterized by the people the child lives with; (2) existence of siblings, from the same or different marital relationship of the parents; (3) relationship between: father/child, mother/child and among siblings, based on the opinion of the caregiver interviewed (good, regular/bad, does not have that relationship); (4) support from close friends/family with whom the caregiver is comfortable to talk [42,43]; (5) external support for the caregiver (church, community, health services, etc.) [42,43]; (6) stressful family life events investigated by financial problems, serious health problem of a family member, relative accused of a crime or in prison, family member with alcohol/drug abuse, parental divorce, remarriage of a parent, and serious disease of child requiring medical care [44]; (7) family violence. *The Conflict Tactics Scale*[45,46] was applied to measure very severe physical violence committed by mother and/or father against the child and intimate partner physical violence. The first is characterized by kicking, biting or hitting, spanking, burning, strangling or suffocating, threatening or using a knife or a gun. To intimate partner physical violence, the same preliminary items were assessed, and include threatening to hit or to throw something between the couple. Both types of violence were rated on a 3-point scale (*never, sometimes/seldom, rarely*). At least one positive answer indicates the victimization of each act of violence. Good Cronbach’s α was found for intimate partner violence (0.82 husband/wife, 0.74 wife/husband) and satisfactory for very severe physical violence by the father and/or mother against the child (0.6). *Sibling violence* was characterized by hurting and/or deprecating the child investigated. *Psychological violence* was investigated through the acts committed by a family against the child studied, such as humiliation, criticism, and use of abusive names such as “crazy,” “idiot,” or “stupid.” Cronbach’s α of 0.71. Almost all of the response scale to the variables used in this block is shown in Table 1.

**Table 1:** Associations of Family Variables with Withdrawn Behavior/Depression in Children, São Gonçalo/RJ/Brazil (intermediate block)

**Family Variables**		**(%) Withdrawn/depression**	**OR**	Confidence Interval*
FAMILY STRUCTURE	Both parents (n = 251)	9.6	-	-
	Without father and mother (n = 17)	5.9	0.59	(0.08-4.66)
	With Stepparent (n = 78)	**21.8**	**2.64**	**(1.32-5.22)**
	With only one of the parents (n = 115)	4.3	0.43	(0.16-1.16)
SIBLING	From same marital relationship (n = 226)	8.4	-	-
	From different marital relationship (n = 205)	11.2	1.37	(0.72-2.61)
	Do not have sibling (n = 32)	18.8	2.51	(0.92-6.86)
FATHER-CHILD RELATIONSHIP	Good (n = 365)	8.8	-	**-**
	Regular/Bad (n = 65)	23.1	2.11	**(1.09-4.09)**
	Do not have (n = 24)	-	-	**-**
MOTHER-CHILD RELATIONSHIP	Good (n = 412)	9.5	-	-
	Regular/Bad (n = 46)	19.6	2.10	(0.95-4.64)
	Do not have (n = 4)	-	-	-
SIBLING RELATIONSHIP	Good (n = 290)	7.9	-	-
	Regular/Bad (n = 134)	14.2	1.92	(1.00.-3.66)
	Do not have (n = 33)	18.2	2.6	(0.96-6.88)
SUPPORT OF CLOSE FRIENDS/FAMILY	Yes (n = 348)	8.9	-	-
	No (n = 94)	16.	1.94	(1.00-3.77)
EXTERNAL SUPPORT	Yes (n = 141)	7.8	-	**-**
	No (n = 301)	11.6	1.55	(0.76-3.16)
FINANCIAL PROBLEMS (stressful life event)	No (n = 193)	5.7	-	**-**
	Yes (n = 267)	14.	2.66	**(1.32-5.36)**
SERIOUS HEALTH PROBLEM (stressful life event)	No (n = 218)	7.3	-	**-**
	Yes (n = 244)	13.	1.91	**(1.01-3.58)**
RELATIVE ACCUSED/PRISON (stressful life event)	No (n = 350)	11.4	-	-
	Yes (n = 110)	7.3	0.61	(0.27-1.34)
ALCOHOL/DRUG ABUSE (stressful life event)	No (n = 293)	8.9	-	-
	Yes (n = 168)	13.1	1.54	(0.84-2.82)
PARENTAL DIVORCE (stressful life event)	No (n = 216)	7.	-	-
	Yes (n = 248)	13.3	2.05	**(1.08-3.9)**
REMARRIAGE OF A PARENT (stressful life event)	No (n = 288)	8.7	-	-
	Yes (n = 175)	13.1	1.59	(0.87-2.9)
CHILD´S DISEASE RECEIVED MEDICAL CARE (stressful life event)	No (n = 247)	8.9	-	-
	Yes (n = 216)	12.	1.4	(0.77-2.55)
VERY SEVERE PHYSICAL VIOLENCE (MOTHER/FATHER X CHILD)	No (n = 427)	9.1	-	-
	Yes (n = 37)	24.3	3.19	**(1.08-1.41)**
HUMILIATION (Psychological Violence)	Rarely/Never (n = 365)	8.2	-	**-**
	Some times (n = 67)	11.9	1.51	(0.66-3.46)
	Always/almost always (n = 30)	30.0	4.78	**(2.01-11.37)**
CRITICISM (Psychological Violence)	Rarely/Never (n = 199)	7.5	-	-
	Some times (n = 188)	9.0	1.22	(0.59-2.52)
	Always/almost always (n = 74)	20.3	3.12	**(1.44-6.76)**
TO CALL OF “CRAZY”, “IDIOT” OR “STUPID” (Psychological Violence)	Rarely/Never (n = 229)	6.6	-	-
	Some times (n = 157)	10.8	1.73	(0.84-3.58)
	Always/almost always (n = 77)	20.8	3.74	**(1.75-7.99)**
INTIMATE PARTNER PHYSICAL VIOLENCE (wife x husband)	No (n = 312)	8.7	-	**-**
	Yes (n = 84)	18.	2.29	**(1.16-4.54)**
INTIMATE PARTNER PHYSICAL VIOLENCE (husband x wife)	No (n = 309)	9.1	-	**-**
	Yes (n = 89)	17.	2.03	**(1.03-4.00)**
SIBLINGS VIOLENCE	No (n = 243)	7.4	-	**-**
	Yes (n = 208)	13.	1.86	(0.99-3.49)

*p < 0.05.

#### Children’s individual variables (proximal level)

The Child Behavior Checklist (CBCL) was applied to evaluate externalizing problem behavior (18 items for aggressive behavior and 17 for rule-breaking behavior) and social competence (children’s activities, hobbies, school performance and sociability - 20-items) [47]. Borderline cases were analyzed in the same category as clinical cases. The version applied was tested on a sample of Brazilian children that demonstrated criterion-validity in distinguishing cases from non-cases when compared with clinical diagnosis [48]. The Externalizing Scale showed good internal consistency (0.95) and correlation with Teacher Report Form [47] (Pearson’s r = 0.25, p < 0.001). Cronbach’s α 0.55 to Social Competence Scale.

#### Childhood depression

The *Withdrawn/Depressed*” subscale of Child Behavior Checklist (CBCL) was applied (8 items) and the T score index proposed for defining the groups: non-clinical (T < 65), borderline (T = 65-69) and clinical (T > 69) [47]. As above, borderline cases were analyzed in the same category as clinical cases. To test criterion-validity, forty-five children were also randomly selected through score comparison between CBCL and K-SADS-PL (Kiddie – Schedule for Affective Disorders and Schizophrenia – Lifetime Version) [49]. Diagnostics performed by two independents child psychiatrists (one following the DSM-IV and another through the KSADS-PL) were compared with those obtained by the CBCL sub-scales. For the *Withdrawn/Depressed*” subscale, the results indicated 100% sensibility and 75% specificity for the correlation of CBCL and DSM-IV, and 100% sensibility and 77% specificity for the correlation between CBCL and KSADS-PL. Cronbach’s α showed internal consistency of 0.82. Brazilian studies have provided support for the multicultural robustness of the CBCL in Brazil [50].

### Data analyses

SPSS 15.0 and R 2.4.1 were used in the analyses. The chi-square test (with or without Yate’s correction for continuity) was used for the bivariate comparison (alpha level of .05), and hierarchical logistic regression analysis to examine the relationship between socio-demographic, family and individual variables with depression [40]. Fisher’s exact test was used in tables with expected cell counts less than 5. The odds ratio and confidence intervals (Wald test) were obtained. The hierarchy consisted of three steps and was structured into the 3 blocks (distal, intermediate and proximal variables). In Likelihood Ratio test type I, an alpha level of .05 was defined *a priori* to elect the variables that would remain in each block of the model studied, since the input of the variables in the levels of analysis (blocks) was performed manually. The quality of the model is informed by the Akaike (AIC) criteria (the lower value indicates the best adjustment).

## Results

### Prevalence and associations with childhood depression

Firstly, 10.3% (CI 7.7-13.2) of all the children sampled were identified as cases of depression by the caregivers. Also, 6% of all the children were identified as victims of very severe physical violence committed by the father and/or mother, 22% of the families experienced severe intimate partner physical violence (committed by one parent against the other), and 47.6% of the informants reported violence among siblings.

With respect to the association of the questions studied with child depression, no socio-demographic block variable investigated proved to be associated (Table 2).

**Table 2:** Associations of social-demographic variables with childhood depression, São Gonçalo/RJ/Brazil (distal block)

**Social-demographic variables**		**(%) Depression**	**OR**	Confidence Interval *
Age of children	6-8 (n = 365)	9.6	-	**-**
	9–10 (n = 99)	13.1	1.42	(0.72-2.81)
Sex of children	Female (n = 224)	9.4	-	-
	Male (n = 240)	11.3	1.22	(0.67-2.23)
Social Stratum	Upper/Middle (n = 210)	11.0	-	(0.61-2.12)
	Poor (n = 179)	12.3	1.140	

*p < 0.05.

Table 1 shows that child depression was significantly associated with families with stepparents, OR 2.6 (CI 1.32-5.22). Similar significant association was verified with child depression and regular/bad relationship with father, OR 2.1 CI (1.09-4.09); and with the following family life events: financial problems OR 2.6 CI (1.32-5.36); serious health problems, OR 1.9 CI (1.01-3.58); and parental divorce, OR 2.1 CI (1.08-3.9).

Interestingly, almost all family violence variables were associated with child depression (Table 1). The main finding was that depression is associated with violence committed by the father and/or mother against the child, OR 3.19 (1.08-1.41). Psychological violence was more common among depressive children that suffered from: humiliation, OR 4.8 CI (2.01-11.37); criticism, OR 3.1 CI (1.44-6.76); and those children that have been called abusive names like “crazy,” “idiot,” or “stupid,” OR 3.7 CI (1.75-7.99). Furthermore, depression was associated with severe intimate partner physical violence committed by wife against husband, OR 2.3 CI (1.16-4.54); and husband against wife, OR 2. CI (1.03-4.00).

Nonetheless, as presented in Table 1, a non-significant tendency toward depression was found in several family variables studied: siblings from the same/different marital relationship of parents; relationship between mother and child, and between siblings; support of close friends/family; external support; relative accused of a crime or in prison; family member with alcohol/drug abuse; remarriage of a parent; serious disease of a child requiring medical care; and sibling violence.

In the proximal block (Table 3), only externalizing behavior was significantly associated with depression, OR 3.9 (CI 2.03-7.59).

**Table 3:** Associations of Individual Variables with Childhood Depression, São Gonçalo/RJ/Brazil (proximal block)

**Individual Variables**		**(%) Withdrawn/depression**	**OR**	Confidence Interval*
SOCIAL COMPETENCE	Non-clinical (n = 312)	9.6	-	-
	Clinical/Borderline (n = 71)	12.7	1.36	(0.61-3.01)
EXTERNALIZING BEHAVIOR	Non-clinical (n = 396)	7.8	-	-
	Clinical/Borderline (n = 68)	25.0	3.9	**(2.03-7.59)**

*p < 0.05.

### Potential psychosocial factors for depression in children

According to the first step in the hierarchical regression analysis, age, sex and social stratification were entered as distal covariates, and non-significant association was verified (p < 0.05). Second, only family covariate information was entered into the model: family structure, relationship between the father and the child investigated, specific stressful family life events (financial problems, serious health problems and parental divorce) and types of family violence (physical violence committed by father and/or mother against the child, psychological violence and intimate partner violence) showed significant association with depression. Third, these significant family variables remained in the model, in addition to the individual proximal covariates. The results of the hierarchical logistic regression analysis are shown in Table 4.

**Table 4:** Final model of Hierarchical Logistic Regression for Withdrawn Behavior/Depression in Children, São Gonçalo/Rio de Janeiro/Brazil (n = 380)

**VARIABLES**		**ODDS RATIO**	**CONFIDENCE INTERVAL (IC95%)**	**AIC (without item)**
**Family**				
Father-Child Relationship	**Regular/Bad**	**3.24**	**(1.32-7.94)**	221.78
	Good	1,00	-	
Humiliation of the child (Psychological Violence)	**Always/Almost always**	**6.13**	**(2.06-18.31)**	224.67
	Some times	0.72	(0.23-2.19)	
	Rarely/Never	1.00	-	
Parental Divorce	**Yes**	**2.89**	**(1.14-7.32)**	220.57
	No	1.00	-	
**Individual**				
Externalizing Behavior Problem	**Borderline/Clinical**	**3.53**	**(1.51-8.23)**	223.53
	Normal	1.00	-	

In the final model, the following variables (Table 4) reveal as potential psychosocial factors for depression in childhood**:** regular/bad relationship with father (OR 3.24, 95% CI 1.32-7.94), psychological violence (humiliation) (OR 6.13, 95% CI 2.06-18.31), parental divorce (OR 2.89, 95% CI 1.14-7.32) and externalizing behavior problems (OR 3.53 IC 1.51-8.23).

## Discussion

The prevalence of depression in childhood (10%) indicated relatively high rates in comparison with other samples using DSM-IV depression diagnosis (from 1% to 8%) [51,52]. However, it is important to note that the prevalence may be overestimated because of the sample characteristics, especially with respect to the nature of the institutions surveyed. Furthermore, these relatively high rates can be explained by the criteria applied for defining the cases of depression and/or by the continuity and variety of risk situations to which most of the children studied are exposed, e.g. poverty, violence, dysfunctional households and difficulty of access to healthcare services. On the other hand, the prevalence verified is equivalent to other studies if only considering clinical cases (6.9%). Using clinical diagnosis, Fleitich-Bilyk & Goodman [4] found moderate to high overall prevalence of psychiatric disorders in Brazilian community children and adolescents (13%), compared to a British survey (10%); however, no difference was found in relation to depressive disorders.

Furthermore, alarming statistics were revealed for family violence, which could also be explained by the social vulnerability of the families investigated, the cultural acceptance of violence in many Brazilian families and the inefficacy of protection services in the country.

With respect to the scope of the study, namely to examine the association of psychosocial factors with depression in childhood, first of all, there is little evidence of depression according to sex and age in childhood. The sex differences are more related to puberty than to chronological age [53]. With regard to the association with socioeconomic status, studies that enclose populations of different social strata can better explain this issue.

In terms of the individual questions block, the association with externalizing behavior can be explained by (1): comorbidity - as Angold & Costello [54] emphasize: ‘it is a real characteristic of the phenomenology of child depressive disorders’ (p.155); and (2): exposure to family violence, which is also a consensual risk factor for aggressive and rule-breaking behaviors.

However, according to other works [12,55-57], all the issues associated with family environment and depression comprise an environment exposed to risk. Parental divorce, a bad relationship between father and child and violence are aspects that are causes of potential depression in childhood. Both factors are interrelated, since there is a tendency for children to remain with the mother after divorce, which may lead to distancing from the father. The feeling of loss, prior or posterior conflicts resulting from separation, fights, and socio-economic aspects are features related to divorce, making the situation even more harmful to the child. The new family organization can facilitate physical and emotional detachment, which reduces family support and induces rejection and hostility.

The finding of a strong link between different types of family violence and child depression, among which psychological violence is highlighted, may indicate that a violent context produces a psychological and emotional imbalance that may trigger the depressive condition. Moreover, it is noteworthy that the low reactive ability of children vis-à-vis depression may contribute to the victimization. Furthermore, the effect of violence can interfere in the prolongation of depression, as the blame, shame, sadness and withdrawal generated by violent situations can all contribute to a depressive constellation that is difficult to revert and foments the condition of victim and depression [58].

These findings can assist clinical decision-making processes by characterizing psychosocial aspects and guiding educators and families. Efforts should focus on public health models for the prevention of violence and on the development of adaptive coping mechanisms, in accordance with the various stages of risk from the developmental perspective. These focal points should be taken into account in early interventions, especially for those children who come from highly vulnerable families. The effects of violence may alter the timing of typical developmental trajectories. Initially, violence may result in depression and externalizing disorders that cause secondary reactions by disrupting the child’s progression through age-appropriate developmental tasks, and consequently, his/her ability to cope with the social world [55].

Lastly, future analyses need to focus on investigating the link between depression and the relationship of the child with the mother and siblings, the support from friends and relatives, and sibling violence, since these issues limit statistical results. Besides that, it is important to understand mediators and protective variables in pathways to depression and to determine whether early interventions with children who are victims of violence can reduce the risk of subsequent depression. Conversely, it can be established whether early intervention with children experiencing this disorder can help to reduce the risk of violent victimization. Moreover, children who were exposed to violence, especially those from an underprivileged background, need to be evaluated and treated by trained clinicians. It is also critical to clarify the understanding of the physiological factors, which may indicate the role of genetic and/or early environmental factors in the origins of depression. An approach that takes into account the combination of psychological, family and physiological factors may contribute to the comprehensive course and outcome of depression through interrelationships with the environment.

### Limitations of the study

The cross-sectional design limits the findings, which should be considered in the interpretation of the results, since it does not permit to investigate the possibility of reverse causality. The access to only one respondent (the caregiver) introduces a limitation, since only one viewpoint is analyzed. Furthermore, the assessments are retrospective, which can introduce a recall bias. Another limitation refers to the variety of measures, which can generate confounding and interaction, though this was partially minimized by block and univariate analysis. Finally, with respect to the psychosocial factors, the majority of them are not specific to any particular disorder; however the identification of potential factors may indicate aspects that must be considered in the prevention and treatment of mental disorders in children.

## Competing interests

The authors declare that they have no competing interest.

## Authors´ contributions

Avanci participated in data collection, conducted the literature search and data analysis, and drafted the article. Assis made a substantial contribution to the methodology and interpretation of results and helped draft the manuscript. Oliveira and Pires were the main people responsible for the data analysis. All of the authors read and approved the final manuscript.
